# Comparative Diagnostic Performance of the Granulocyte and Neutrophil Counts

**DOI:** 10.1016/j.plabm.2017.10.001

**Published:** 2017-10-04

**Authors:** Nicola S. Pether, Jessica L. Brothwood, Cornelis van Berkel, Elaine H. Dunwoodie, Robert L. Blake, Christopher P. Price, Richard G. Jones, Karl S. Baker, Geoff Hall

**Affiliations:** a Institute of Health Science, the University of Leeds, UK; b Leeds Teaching Hospitals NHS Trust, Leeds, UK; c Philips Electronics UK Ltd., UK; d Leeds Institute of Cancer and Pathology, University of Leeds, UK; e Nuffield Department of Primary Care Health Sciences, University of Oxford, UK

**Keywords:** Cancer, Haematology analyser, Neutropenia, Neutrophil count, Granulocyte count, Diagnostic performance

## Abstract

**Objectives:**

Use of point-of-care testing is increasing, however many haematology analysers can only determine granulocyte count without further differentiation into neutrophils, eosinophils and basophils. Since the diagnosis of life-threatening neutropenia in cancer patients requires a distinct neutrophil count, this study aimed to determine the comparative performance between the neutrophil and granulocyte count.

**Design and methods:**

A database of 508 646 venous full blood count results measured on a laboratory reference analyser was mined from a large oncology unit. The relationship between granulocyte and neutrophil counts was assessed. Multinomial logistic regression was used to classify results into neutropenia grades using an equivalent granulocyte count.

**Results:**

Granulocyte to neutrophil count correlation was 0.997. The accuracy for classification into neutropenia grades using the derived equivalent granulocyte count ranges was 96.4%. Identification of results with a neutrophil count <1.5×10^9^ cells/L using an equivalent granulocyte count of <1.69×10^9^ cells/L resulted in sensitivity, specificity, positive and negative predictive values of 98.0%, 99.5%, 97.8% and 99.5%, respectively.

**Conclusions:**

These results describe the relationship between granulocyte and neutrophil counts, measured on a laboratory analyser, in a large population of patients with malignancies and receiving anti-cancer therapies. However, this relationship must be established using a point of care testing system with a three-part differential count before considering the possibility that a granulocyte count can guide clinical decisions in the absence of a definitive neutrophil count, to reduce the frequency and severity of neutropenic complications in patients receiving cancer treatments.

## 1 Introduction

The technology of morphological assessment and counting of blood cells has advanced over recent decades, particularly in the white cell lineage, with concomitant benefits in relation to diagnosis, prognosis and management of inflammatory and malignant conditions. The full range of measurements is available on modern automated laboratory analysers. However, dependence on a service provided by a central laboratory has certain limitations, with potential clinical, operational and economic implications. These issues could arise in any setting where rapid decision making is required, e.g. the Emergency Department, primary care, a paramedical rural service, an out-of-hours doctor service or in the home, as well as in middle- and low-income countries [1], [2], [3], [4], [5].

One of the major technological advances has been in the recognition and quantification of differential white count. The initial three-part differential count expanded to the five-part differential count, with differentiation of the granulocyte count into neutrophil, basophil and eosinophil counts. Viral and bacterial infections are arguably the most common cause of acquired neutropenia [6], [7], through margination of neutrophils and destruction by circulating antibodies [8]. Neutrophil levels may also be decreased due to congenital haematological malignancies [9], as a result of radiotherapy [10] or through the use of cytotoxic chemotherapy drugs [11]. Extreme reductions in neutrophil count can lead to serious complications such as febrile neutropenia (fever >38°C and neutrophil count <0.5×10^9^cells/L [12]), increasing the risk of sepsis-associated mortality [13], necessitating urgent clinical assessment in at risk patients. Thus, most chemotherapy patients are given immediate empirical antibiotics upon suspicion of infection [14]. In such patients, access to a rapid differential white count is vital as delays in administration of broad-spectrum intravenous antibiotics are associated with increased mortality risk, but overtreatment with unnecessary antibiotics has opportunity costs [15].

Access to the absolute neutrophil count (ANC) can be difficult in the early phase of developing neutropenia in patients on chemotherapy. These patients tend to be at home, and a health-care professional is required to obtain a venous sample from frequently accessed veins which need to be preserved for delivery of chemotherapy, but are often already compromised by vesicant and irritant cytotoxic drugs. Thus, it is not routine practice to monitor the neutrophil count during the chemotherapy cycle unless the patient reports symptoms suggestive of developing severe neutropenia complicated by infection.

There have been very few studies comparing the diagnostic performance of granulocyte and neutrophil counts in patients receiving chemotherapy. The aims of this study were to (i) determine the threshold of total granulocytes which represents a neutrophil count which signals a change in patient management, and (ii) determine if total granulocytes could be used as a meaningful indicator of neutrophil count in the neutropenic range for cancer patients receiving chemotherapy. This study was the first step in determining whether it was valid to consider the use of a granulocyte count for monitoring patients receiving chemotherapy.

## 2 Material and methods

### 2.1 Study design and patient selection

Analysis was conducted on a pseudonymised, retrospective database containing peripheral venous blood sample results between 1 January 2004 and 1 September 2013 from 21,020 patients, all of whom had received chemotherapy treatment at the Leeds Cancer Centre, Leeds Teaching Hospitals Trust (LTHT). The LTHT results server receives blood test results from the pathology laboratories and displays them in the electronic patient recording system (Patient Pathway Manager (PPM)) [16], [17]. A pseudonymised extract was taken and inserted into a research database. No identifiable data was contained within the dataset and the research was sanctioned under the information governance procedures of LTHT, with data extraction pseudonymisation procedures as agreed with the Caldicott Guardian and with formal approval from a national research ethics committee (NHS Grampian ID: 13/NS/0128). No patients were excluded based on their chemotherapy treatment, demographic information, diagnosis or timing of treatment.

Blood counts were measured from EDTA venous whole blood samples obtained for the purposes of routine clinical care, and taken at any time in relation to chemotherapy delivery. All samples were submitted for a full blood count analysis, including a five-part differential on a Siemens ADVIA 120 analyser (Siemens Healthcare Diagnostics, Erlangen, Germany) until August 2004 and subsequently on the Siemens ADVIA 2120 analyser; both instruments employ the same method principles. All instruments were subjected to multiple quality control (QC) checks each day according to standard laboratory protocols, and the laboratory participated in the United Kingdom National External Quality Assessment Service (UKNEQAS) external quality assurance scheme.

Data of interest included the eosinophil, basophil and neutrophil counts, with the sum of these three parameters being taken as the granulocyte count (calculated in Microsoft SQL Server). Lymphocyte and monocyte results were also extracted for analysis. As within-day timing information was not available, if a patient had more than one blood test on a given day all data for that day was excluded to avoid ambiguity as to which result should be taken as the true value for that day.

### 2.2 Correlation and regression analysis

The R programming language package was used to conduct all statistical analysis and produce all figures [18]. Pearson's product-moment correlations were used to measure the strength of the linear association between complete granulocyte count and each of its components (eosinophils, basophils and neutrophils); *p* < 0.05 was considered significant. To correct for the differences in scale, raw count data was log transformed and standardized (x′= [ln {x}- mean (ln {x})]/ standard deviation (ln{x}). Passing-Bablok regression analysis was conducted using the MCR package for R [19]. This was performed separately on subsets of individuals with neutrophil counts classified as N0-N1 (normal to grade 1 neutropenia, ≥ 1.5 to ≤ 7.5 × 10^9^ cells/L) and N2-N4 (grade 2–4 neutropenia, < 1.5 × 10^9^ cells/L) using grading criteria defined by The Common Terminology Criteria for Adverse Events [20]. To limit the memory requirements and computational overhead, the regression analysis was on a random subset of 32,000 results in each subset.

### 2.3 Difference analyses

Bland-Altman plots were constructed in order to assess the relation between neutrophil and total granulocyte count [21] where the difference between measures is plotted against the average of the two measurements. Good concordance can be concluded if enough points fall within narrow limits of agreement, to be confident that one method could be used in the place of another i.e., the mean difference should be close to zero and at least 95% of differences should not exceed 1.96 standard deviations (SD).

### 2.4 Classification into neutropenia grades

The data was divided using random split sampling (1:2) into derivation and validation datasets. Multinomial logistic regression using the VGAM package [22] was employed on the derivation data to derive equivalent granulocyte count ranges to classify each neutrophil result by neutropenia grade (as defined above). Model performance measures were reported for the validation dataset at each neutrophil classification section grade and it was also assessed on its ability to identify N2–N4 neutrophil results. Finally this threshold was adjusted using optimised values for specific objectives using the ‘Optimal Cutpoints’ package [23].

## 3 Results

### 3.1 Data distribution

There were 508,646 test results with only one neutrophil, eosinophil, basophil, monocyte and lymphocyte count result per patient per day. The distribution of count results for complete granulocyte and each of the differential counts was assessed (Fig. 1). The total number of results within the reference range was 258,363 (50.8%) for neutrophils (2.5–7.5 × 10^9^ cells/L), 329,179 (64.7%) for eosinophils (0.04–0.4×10^9^ cells/L) and 436,970 (85.9%) for basophils (0.01–0.1 × 10^9^ cells/L). In total, 187,003 (36.8%) results fell within the reference range for eosinophil, basophil and neutrophil results and there were 404,935 (79.6%) results within the upper limit of normal for all three granulocyte components. When considering granulocytic disease states, 172,266 (33.9%) of results had neutropenia (< 2.5 × 10^9^ cells/L), 78017 (15.3%) neutrophilia (> 7.5 × 10^9^ cells/L), 158,353 (31.1%) eosinopenia (< 0.04 × 10^9^ cells/L), 21,114 (4.2%) eosinophilia (> 0.4 × 10^9^ cells/L), 50,311 (9.9%) basopenia (< 0.01 × 10^9^ cells/L) and 21,365 (4.2%) basophilia (> 0.1 × 10^9^ cells/L).

**Fig. 1:**
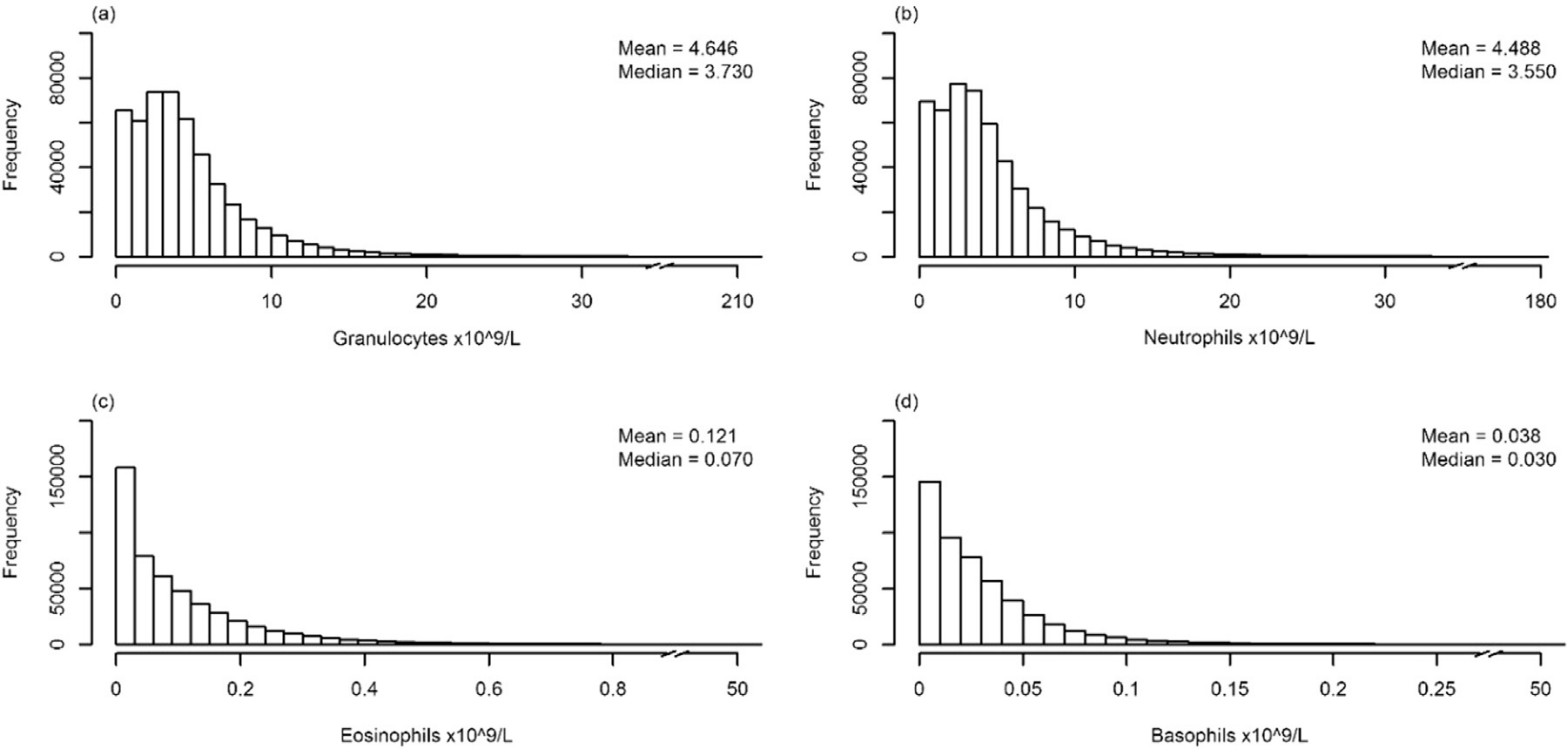
Distribution of cell count results for total granulocytes and individual differentials. Histograms of 508646 results for (A) granulocytes (x 10^9^ cells/L) (minimum = 0; maximum = 213.42, median = 3.73; mean = 4.65; standard deviation (SD) = 4.31; (B) neutrophils (x 10^9^ cells/L) (minimum = 0; maximum = 180.58, median = 3.55; mean = 4.49; SD = 4.21); (c) eosinophils (x 10^9^ cells/L) (minimum = 0; maximum = 53.69, median = 0.07; mean = 0.12; SD = 0.26); (d) basophils (x 10^9^ cells/L) (minimum = 0; maximum = 51.43, median = 0.03; mean = 0.04; SD = 0.15).

### 3.2 Correlation and regression

It is acknowledged that neutrophils are the largest component of granulocytes; however, the extent of this relationship and that to the other differentials was investigated with a view to predicting neutrophil count. Correlation coefficients compared to the neutrophil count were found to be 0.997 for granulocytes (R^2^ = 0.995), 0.203 for eosinophils (R^2^ = 0.041), 0.248 for basophils (R^2^ = 0.062) and 0.266 for eosinophils plus basophils (R^2^ = 0.071). To lessen the effects of skew and differences in scale, analysis was also conducted using standardised natural logarithmically transformed values. Although correlations improved upon transformation, overall they remained weak for eosinophils and basophils (0.998 for granulocytes, 0.405 for eosinophils and 0.452 for basophils).

**Fig. 2:**
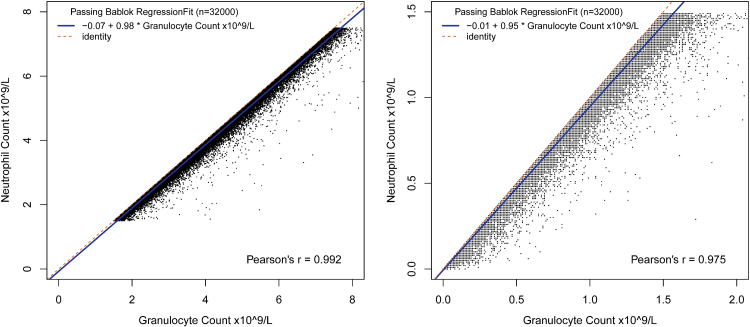
Relationship of neutrophil and granulocyte counts for (a) normal and neutropenic grade 1 (neutrophil count 1.5–7.5×10^9^cells/L), (b) neutropenic grades 2–4, neutrophil count < 1.5 × 10^9^cells/L).

Passing-Bablok regression between granulocyte count and neutrophil count was performed in two groups according to neutrophil result: N0-N1 (normal or grade 1 neutropenia, neutrophils 1.5–7.5 × 10^9^ cells/L, *n* = 331977) and N2-N4 (grade 2–4 neutropenia, neutrophils < 1.5 × 10^9^ cells/L, *n* = 98652). Fig. 2 illustrates both results. All data pairs lie necessarily below the identity line and are in fact highly concentrated around the regression line, with some spread of outliers below the regression line. Slopes and intercept with confidence intervals are summarized in Table 1.

**Table 1:** Slopes and intercepts, and their respective confidence intervals, from the Passing-Bablock regression of granulocyte and neutrophil counts, grouped according to neutropenia grades.

	Intercept	CI	Slope	CI
N0-N1	−0.071	−0.074, −0.068	0.984	0.983, 0.985
N2-N4	−0.0063	−0.0068, −0.006	0.954	0.953, 0.955

### 3.3 Difference analyses

**Fig. 3:**
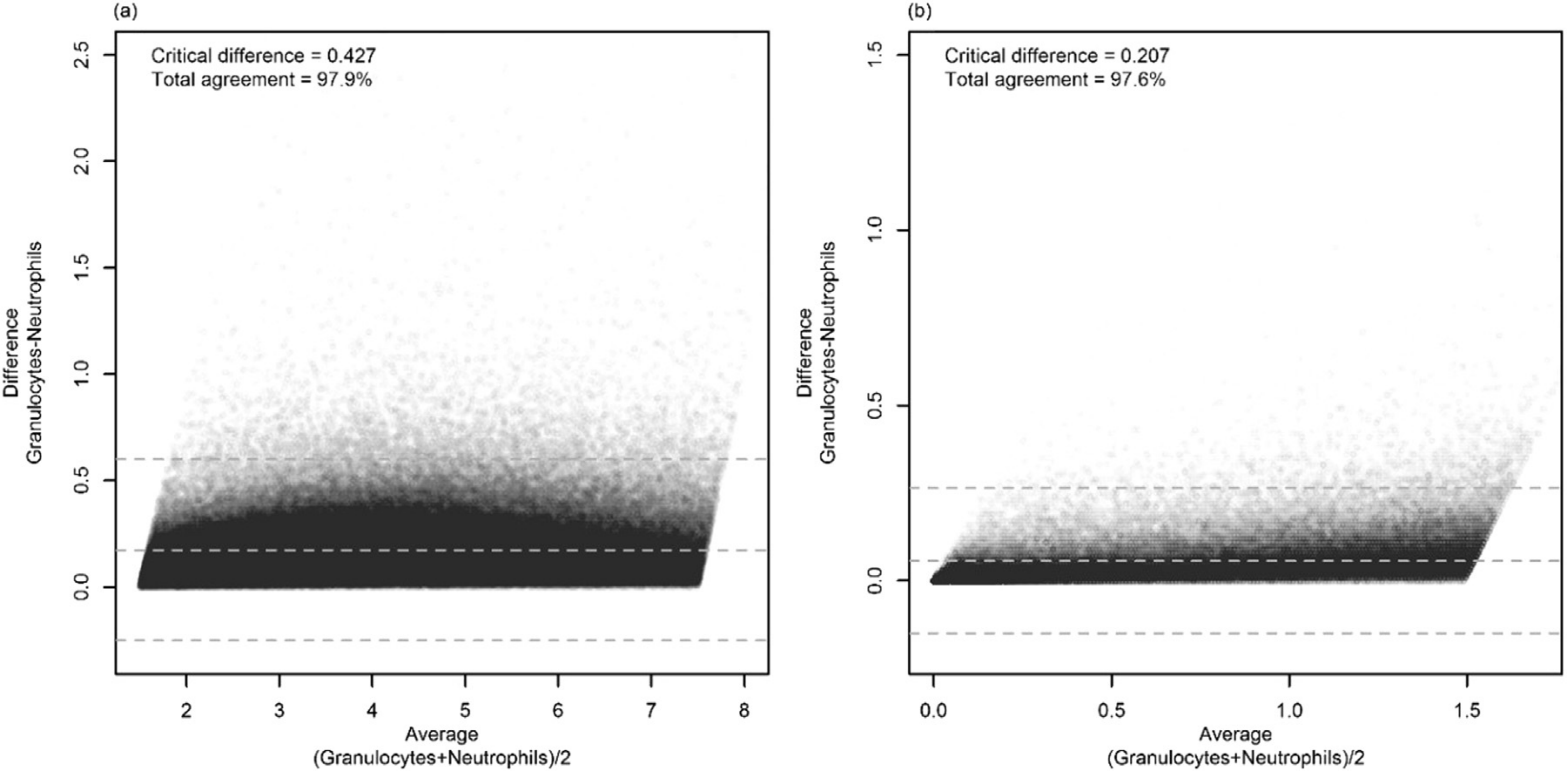
Differences analysis for (A) normal and neutropenic grade 1 and (B) neutropenic grade 2–4 results. Bland-Altman plots showing (A) N0-N1 results (neutrophil count 1.5–7.5×10^9^cells/L), *n* = 331977 and (B) N2-N4 results (neutrophil count <1.5 × 10^9^cells/L), *n* = 98652. Grey dashed lines from top to bottom: upper limit of agreement (+1.96 SD) (A) 0.600, (B) 0.265; average difference (A) 0.174, (B) 0.058; lower limit of agreement (−1.96 SD) (A) −0.252, (B) −0.149; critical difference (A) 0.427, (B) 0.207. Note the lower limits are redundant since difference cannot be less than 0. Points plotted with a transparency alpha of 0.01.

As previously, counts were split into N0-N1 and N2-N4 results. Fig. 3A shows that 97.9% of results had a granulocyte count not exceeding the neutrophil count by more than 0.600 × 10^9^ cells/L (the upper limit of agreement). In other words, the sum of only 7048 out of 331,977 eosinophil and basophil counts exceeded 0.600 × 10^9^ cells/L. Investigation of the N2–N4 results revealed only 2338 results out of 98652 (2.4%) had a granulocyte count exceeding the neutrophil count by more than 0.265 × 10^9^ cells/L (Fig. 3B). Analysis on individual neutropenia grades revealed 97.0% of grade 1 results had a difference of less than 0.358×10^9^ cells/L (mean 0.111), 96.6% of grade 2 results were < 0.278 × 10^9^ cells/L (mean 0.086), 97.2% of grade 3 results were < 0.242 × 10^9^ cells/L (mean 0.066) and 97.5% of grade 4 results < 0.140 × 10^9^ cells/L (mean 0.026).

### 3.4 Classification

Agreement analysis found a relatively small mean difference for both the N0-N1 and N2–N4 neutrophil result ranges. Therefore, the ability to correctly classify neutropenia grades using an equivalent granulocyte count range was investigated. The complete dataset was randomly split (1:2) into derivation (*n* = 167,853) and validation (*n* = 340,793) subsets on which a multinomial logistic regression classifier was trained and tested, respectively (Supplemental data, Fig. 1).

An equivalent granulocyte range was derived by the classifier for each neutrophil determined grade (Table 2), achieving an accuracy of 96.4%. Classification of results within the reference range using granulocyte counts between 2.64 and 7.72 × 10^9^ cells/L was correct 98.1% of the time but incorrectly included a grade 1 neutropenia result in 1.2% of cases. The equivalent grade 1 count (1.6–2.63 × 10^9^ cells/L) was correct for 93.0% of its predictions but had a 2.6% chance of misclassifying a grade 2 neutropenia result. The worst performance, positive predictive value (PPV) of 88.5%, occurred when identifying grade 2 neutropenia (1.08–1.60 × 10^9^cells/L) with a 4.9% chance of identifying grade 3 neutropenic patients and 2.7% displaying grade 4 neutropenia. Identification of grade 4 neutropenia using a granulocyte cut-off at 0.56 × 10^9^cells/L resulted in a PPV of 97.5% but had a 2.5% chance of including a grade 3 neutropenic patient.

**Table 2:** Classification of the validation dataset (*n* = 340793) into neutrophil grades using an equivalent granulocyte count.

Grey boxes indicate correctly identified results. Sn, sensitivity; Sp, specificity; PPV, positive predictive value; NPV, negative predictive value.

In addition to the multinomial classification above, we also investigated binary classification with single granulocyte count boundaries.

The ability to correctly distinguish between N0-N1 results (1.5–7.5 × 10^9^ cells/L) and N2-N4 neutropenia (< 1.50 × 10^9^ cells/L) was assessed using various cut points. Maximising the product of sensitivity and specificity resulted in a threshold of < 1.69 × 10^9^ cells/L, and excellent discriminatory performance (Table 3), but 862 patients with a neutrophil count <1.50×10^9^ cells/L were missed using this selection criterion. To achieve 100% sensitivity a threshold of < 2.39 × 10^9^cells/L could be used, missing only 49 of the 98652 patients with ANC < 1.50 × 10^9^cells/L, but this is accompanied by a decrease in specificity (86.5%) and PPV (64.1%), mistakenly including 55164 patients with ANC ≥ 1.50 × 10^9^ cells/L (10.8%). Use of a granulocyte count threshold of < 1.53 × 10^9^ cells/L results in 100% specificity, mistakenly including only 129 of the 508646 patients with ANC ≥ 1.50 × 10^9^ cells/L, but predicting 4402 as normal/grade 1 neutropenia when they were more severely neutropenic (sensitivity 95.5%, PPV 99.9%, negative predictive value (NPV) 98.9%).

**Table 3:** Classification of neutropenic results using various granulocyte count thresholds.

**Granulocyte Threshold (x10**^**9**^**cells/L)**		**TP (*****n*****)**	**FP (*****n*****)**	**TN (*****n*****)**	**FN (*****n*****)**	**Sn (%)**	**Sp (%)**	**PPV (%)**	**NPV (%)**
**Grade 2–4**	i) **<1.53**	94250	129	409865	4402	95.5	100.0	99.9	98.9
	ii) **<1.69**	97790	6849	403145	862	99.1	98.3	93.5	99.8
	iii) **<2.39**	98603	55164	354830	49	100.0	86.5	64.1	100.0
**Grade 3–4**	i) **<1.03**	66281	195	439569	2601	96.2	100.0	99.7	99.4
	ii) **<1.13**	68155	3436	436328	727	98.9	99.2	95.2	99.8
	iii) **<1.71**	68848	37051	402713	34	100.0	91.6	65.0	100.0
**Grade 4**	i) **<0.51**	42703	38	463844	2061	95.4	100.0	99.9	99.6
	ii) **<0.62**	44361	3359	460523	403	99.1	99.3	93.0	99.9
	iii) **<1.15**	44742	28437	435445	22	100.0	93.9	61.1	100.0

TP, true positive; FP, false positive; TN, true negative; FN, false negative; Sn, sensitivity; Sp, specificity; PPV, positive predictive value; NPV, negative predictive value; n = 508646. Decision points chosen maximise (i) specificity (ii) the product of sensitivity and specificity (iii) sensitivity.

The separation of results based on different grade boundaries was also investigated (Table 3). Using the product of sensitivity and specificity, capture of grade 3 neutropenia results (< 1.0 × 10^9^ cells/L) can be achieved using a granulocyte count of < 1.13 × 10^9^ cells/L to provide sensitivity of 98.9%, specificity of 99.2%, PPV of 95.2% and NPV of 99.8%. Grade 4 neutropenia results can be discriminated using a threshold of < 0.62 × 10^9^cells/L to give sensitivity 99.1%, specificity 99.3%, PPV 93.0% and NPV 99.9%.

### 3.5 Dataset restrictions

Exclusion of patients with eosinophilia (≥ 0.4 × 10^9^ cells/L) or basophilia (≥ 0.1 × 10^9^ cells/L) from the dataset (*n* = 469433) improves the correlation of granulocytes to neutrophils with resulting R^2^ = 0.996 for the N0-N1 results and 0.988 for N2–N4 neutropenic patients. Agreement analysis resulted in 94.4% agreement with a mean difference of 0.192 (upper limit 0.335) and 94.9% agreement with a mean difference of 0.122 (upper limit 0.174).

When the dataset is restricted to include only patients that have received cytotoxic chemotherapy within 42 days (*n* = 279992), the correlation again improves with R^2^ = 0.989 and 0.978 for N0–N1 and N2–N4 neutropenia patients, respectively. Furthermore, Bland-Altman plots showed 97.1% agreement with a mean difference of 0.321 (upper limit 0.464) and 96.9% agreement at 0.164 (upper limit 0.216) respectively.

## 4 Discussion

A good correlation between granulocyte and neutrophil counts was observed, with a linear regression line almost identical to the line of identity. This was maintained even when the neutrophil count was reduced to less than 1.5 × 10^9^ cells/L, which is the reference range most relevant to decision points in oncological practice. The Bland-Altman analysis indicated good agreement between granulocyte and neutrophil counts with 97.6% of granulocytes being within 0.265 × 10^9^ cells/L of neutrophil counts when all neutrophil counts were less than 1.5 × 10^9^ cells/L. Furthermore, we presented the first definition of granulocyte counts equivalent to CTCAE neutropenic grades.

In clinical practice there would be little need to change management decisions based on the specific grade of neutropenia, but, more likely, on which side of a specified threshold the patients’ neutrophil count falls. Therefore, we investigated the boundaries of clinically relevant thresholds, identifying that if <1.5 × 10^9^ cells/L neutrophils is used, the best performing granulocyte count would be <1.69 × 10^9^ cells/L, and if <1.0 × 10^9^/L neutrophils is used, the best performing granulocyte count would be <1.13 ×10^9^ cells/L. Both of these scenarios had a NPV of 99.8% which translates into only 1 in 500 results which would be misclassified as above the threshold.

International guidelines for the treatment of neutropenic fever recommend an ANC <0.5 ×10^9^ cells/L as the threshold for change in clinical management [24], [25], [26], [27], [28], [29]. However, it should be noted that these guidelines assume a full clinical assessment is carried out. Three-part differential analysers might have the potential to be used as point of care devices where the patient may be remote from the clinician; therefore, this work analysed the performance of granulocytes with reference to neutrophil count thresholds greater than 0.5 × 10^9^/L to allow for safety margins required due to absent clinical information. The important clinical question is: if using the equivalent granulocyte count indicates the patient has an ANC above the specified threshold, how sure can we be that this is correct? As an example, using granulocyte count to indicate a neutrophil result is less than 1.0 × 10^9^/L, the best performing granulocyte count in terms of balancing false negatives with false positives would be <1.13 × 10^9^/L, but in order to obtain no false negative results, a significantly higher threshold needs to be used. This would be offset by increasing the false positives. It is a clinical decision as to whether this is acceptable, and is dependent upon the consequences of misclassification of a result. However, this concept would have to be tested in an appropriate clinical trial setting with a point of care testing (POCT) system with a 3-part differential measurement capability.

The strength of this data is the large cohort of patients employed in this analysis, with a wide variety of patients in terms of their neoplasm diagnosis, other co-morbidities, demographic information and many possible neutrophil abundancies. All grades of neutropenia are represented and this may be due to cytotoxic treatments such as chemotherapy or the cancer diagnosis itself.

However, these findings must be interpreted with considerable caution and there are a number of limitations that must be taken into account when drawing conclusions from this work. Firstly, the methodology of granulocyte extraction by summating its differentials is not a direct result from a haematology analyser and therefore may underestimate errors between readings. Secondly, both measurements were made on a ‘state-of-the art’ haematology analyser in a central laboratory, albeit as part of routine daily practice. Thirdly, there are recognised differences in the quantitation of individual white cell species with the use of different detection technologies. This means that variation in the counting technology due to cell population differences may impact on the results, e.g., in the presence of blast or immature granulocytes or neutrophils.

We suggest it is technically feasible to use granulocytes, but this should always be after a baseline reference analyser neutrophil count, to exclude patients at high risk of misclassification on the wrong side of a specified decision threshold. Such high-risk patients would include those with eosinophilia or basophilia, or active allergic conditions.

This study has considered the single result in the context of decision making; it is possible that such measurements may be used for routine monitoring. We conclude from this large cohort of data that the granulocyte count warrants further consideration as a surrogate indicator of neutrophil count. It could be used to indicate the CTCAE grade of neutropenia and its use could be considered in patients with suspected febrile neutropenia, where an alternative neutrophil count may not be readily available. However, it must be recognised that this relationship has been established with both measurements made on an established laboratory analyser system. This relationship cannot be assumed to be applicable to laboratory analysers employing different detection methodologies or to POCT systems. Therefore, the findings from this study cannot be extrapolated to the POCT situation. If POCT, using a three-part differential white count, is to be considered for monitoring patients on chemotherapy away from the hospital setting, then the relationship between the granulocyte and neutrophil count must be established using the technologies that will be used in routine practice [30], [31], [32]; with an appropriate cohort of patients exhibiting the range of neutrophil counts expected to be experienced, and as a precursor to a clinical trial.

## Figures and Tables

**Fig. 1:**
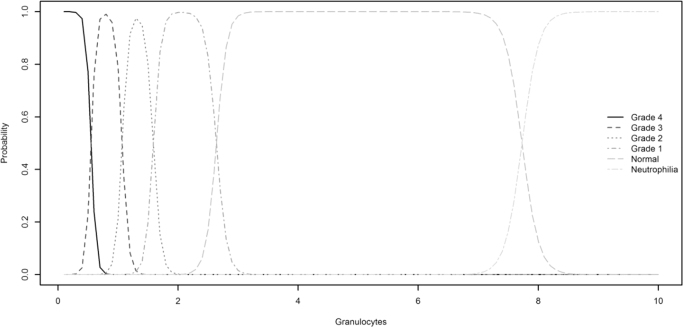
Neutropenia grading probabilities produced by the multinomial logistic regression classifier on granulocyte counts from 0.1 to 10×10^9^cells/L.
